# Impact of Hypokalemia on Electromechanical Window, Excitation Wavelength and Repolarization Gradients in Guinea-Pig and Rabbit Hearts

**DOI:** 10.1371/journal.pone.0105599

**Published:** 2014-08-20

**Authors:** Oleg E. Osadchii

**Affiliations:** 1 Department of Biomedical Sciences, University of Copenhagen, Copenhagen, Denmark; 2 Department of Health Science and Technology, University of Aalborg, Aalborg, Denmark; The Ohio State University, United States of America

## Abstract

Normal hearts exhibit a positive time difference between the end of ventricular contraction and the end of QT interval, which is referred to as the electromechanical (EM) window. Drug-induced prolongation of repolarization may lead to the negative EM window, which was proposed to be a novel proarrhythmic marker. This study examined whether abnormal changes in the EM window may account for arrhythmogenic effects produced by hypokalemia. Left ventricular pressure, electrocardiogram, and epicardial monophasic action potentials were recorded in perfused hearts from guinea-pig and rabbit. Hypokalemia (2.5 mM K^+^) was found to prolong repolarization, reduce the EM window, and promote tachyarrhythmia. Nevertheless, during both regular pacing and extrasystolic excitation, the increased QT interval invariably remained shorter than the duration of mechanical systole, thus yielding positive EM window values. Hypokalemia-induced arrhythmogenicity was associated with slowed ventricular conduction, and shortened effective refractory periods, which translated to a reduced excitation wavelength index. Hypokalemia also evoked non-uniform prolongation of action potential duration in distinct epicardial regions, which resulted in increased spatial variability in the repolarization time. These findings suggest that arrhythmogenic effects of hypokalemia are not accounted for by the negative EM window, and are rather attributed to abnormal changes in ventricular conduction times, refractoriness, excitation wavelength, and spatial repolarization gradients.

## Introduction

The electromechanical (EM) window refers to the time difference between the end of ventricular contraction and the end of the QT interval on ECG. In healthy human subjects, the duration of mechanical systole is greater than the duration of ventricular repolarization, thus contributing to the positive EM window [1]–[2]. These relationships, however, are reportedly reversed in the setting of cardiovascular disease [3]–[5], which may be associated with either QT interval lengthening or shortened ventricular systole, or both. Collectively, these changes lead to the negative EM window, wherein the QT interval exceeds the duration of ventricular contraction. The negative EM window is thought to facilitate the arrhythmic events, because the relatively long electrical systole (i.e., the QT interval) is associated with increased time for Ca^2+^ entry and increased propensity to spontaneous sarcoplasmic reticulum Ca^2+^ release; these changes may promote after-depolarizations, which act as triggers for tachyarrhythmia [6]–[9]. Accordingly, the negative EM window has been recently proposed as a novel preclinical marker of increased propensity to ventricular tachyarrhythmia (VT), especially in the setting of drug-induced prolongation of repolarization [6], [8]–[10]. The measurements of the EM window value, therefore, may be potentially important in cardiovascular safety studies dealing with compounds which prolong the QT interval, for example, by blocking the rapid and/or slow components of the delayed rectifier K^+^ current (*I*_Kr_ and *I*_Ks_, respectively), or increasing the late sodium current (*I*_Na-L_) [11]–[13].

Cardiovascular disease is often associated with reduced serum K^+^ concentrations, which represents a side effect of diuretic therapy. Hypokalemia provokes QT interval lengthening, and markedly increases arrhythmogenic risks in cardiac patients [14]–[17]. The purpose of the present study was to determine whether the proarrhythmic effects of hypokalemia may be partly accounted for by the reversed relationships between the duration of mechanical systole and QT interval, which results in the negative EM window. The proarrhythmic effects of hypokalemia and contributing electrophysiological changes were assessed in whole perfused hearts from guinea-pigs and rabbits, two clinically relevant animal models that are commonly used to study cardiac arrhythmia associated with prolonged repolarization [18]–[19]. The changes in EM window in hypokalemic hearts were examined in parallel with more conventional electrophysiological assessments, including measurements of ventricular conduction times, refractoriness, excitation wavelength index, and spatial repolarization gradients.

## Materials and Methods

This study complies with the European Community Guidelines for the Care and Use of Experimental Animals, and was approved by the Animal Ethics Screening Committee of the Panum Institute (clearance number: 2010/561-1799). Female Dunkin-Hartley guinea-pigs (supplied by Charles River, Sulzfeld, Germany; body weight 400–500 g) and female New Zealand white rabbits (supplied by Lidkoping Kanin Farm, Lidkoping, Sweden; body weight 3.5–4.0 kg) were allowed to acclimate to the housing conditions, with free access to food and tap water, for at least 7 days prior to entry into the study.

### Isolated, Langendorff-perfused heart preparations

The experiments on isolated, perfused hearts were performed as described previously [20]–[21]. The guinea-pigs were anesthetized with sodium pentobarbital (50 mg/kg i.p.), and the rabbits were anesthetized with a mixture of ketamine and xylazine (70 and 10 mg/kg, respectively, i.m.). The animals were anticoagulated with heparin (1000 IU/kg i.p.). Thereafter, the chest was opened, and the hearts were immediately excised and mounted on a Langendorff perfusion set-up (Hugo Sachs Elektronik-Harvard Apparatus GmbH, March-Hugstetten, Germany). The hearts were perfused via the aorta at a constant pressure (60 mm Hg) with carefully filtered, warmed physiological saline solution saturated with 95%O_2_ and 5%CO_2_. During control normokalemic perfusions, the perfusion solution contained (in mM) 118.0 NaCl; 4.7 KCl; 2.5 CaCl_2_; 25 NaHCO_3_; 1.2 KH_2_PO_4_; 1.2 MgSO_4_; and 10.0 glucose, and had a pH of 7.4. Hypokalemic perfusions were accomplished using 2.5 mM K^+^-containing solution.

The Langendorff perfusion set-up was equipped with two ISOTEC pressure transducers (Harvard Apparatus, Holliston, MA, USA) to measure aortic pressure and left ventricular (LV) developed pressure. The aortic pressure transducer was connected to the aorta block of the set-up. The ventricular pressure transducer was coupled to the balloon-tipped catheter introduced into the LV cavity via an incision made in the left atrium. The volume of the LV balloon was adjusted to yield an end-diastolic pressure of 0–5 mm Hg. LV developed pressure was calculated as the difference between end-systolic and end-diastolic pressure. The coronary flow rate was determined using an ultrasonic flowmeter probe (Transonic Systems Inc., Ithaca, NY, USA) placed just above the aortic cannula. The electrical activity of the heart preparations was assessed from the volume-conducted ECG as well as monophasic action potential recordings.

Throughout the experiments, the heart preparations were kept immersed in the temperature-controlled, perfusate-filled chamber to minimize thermal loss. LV developed pressure, aortic pressure, coronary flow rate, ECG and ventricular monophasic action potentials were continuously monitored using a 16-channel PowerLab system (ADInstruments, Oxford, UK).

### Ventricular stimulation and electrophysiological recordings

Previous works suggest that hypokalemia predominantly increases VT inducibility in the LV rather than the RV chamber [22]. Hence, in the present study, the pacing stimulation protocol was applied at the LV epicardial base. The heart was continuously paced (S_1_–S_1_ interval: guinea-pig = 250 ms, rabbit = 350 ms) using 2 ms rectangular pulses of twice the diastolic threshold current generated by a programmable stimulator (Hugo Sachs Electronik-Harvard Apparatus GmbH, March-Hugstetten, Germany). A premature extrastimulus (S_2_) was delivered at 10 s intervals to the pacing site, progressively reducing the S_1_–S_2_ coupling interval in 5–10 ms steps from 150 ms until the preparation failed to respond to S_2_. The effective refractory period was defined as the longest S_1_–S_2_ interval producing no extrasystolic response.

Monophasic action potentials (MAP) were recorded using three LV and three RV pressure contact electrodes applied to the basal epicardial surface (Figure S1). The MAP duration was measured at 90% repolarization (APD_90_). The local activation times were measured at the LV and RV MAP recording sites as the intervals between the pacing stimulus and the fastest upstroke of the following action potential. Ventricular repolarization time was found as the sum of the activation time and the corresponding APD_90_ value. Transepicardial variability of repolarization time was assessed by calculating the standard deviation of the mean repolarization time value determined from six MAP recording sites.

During LV epicardial pacing, the LV-to-RV conduction time was determined as the difference between the mean RV and LV activation times, which were obtained from three MAP recording sites within each ventricular chamber. The excitation wavelength index was calculated as a ratio of effective refractory period and the LV-to-RV conduction time [23]–[25].

### Electromechanical window

**Figure 1:**
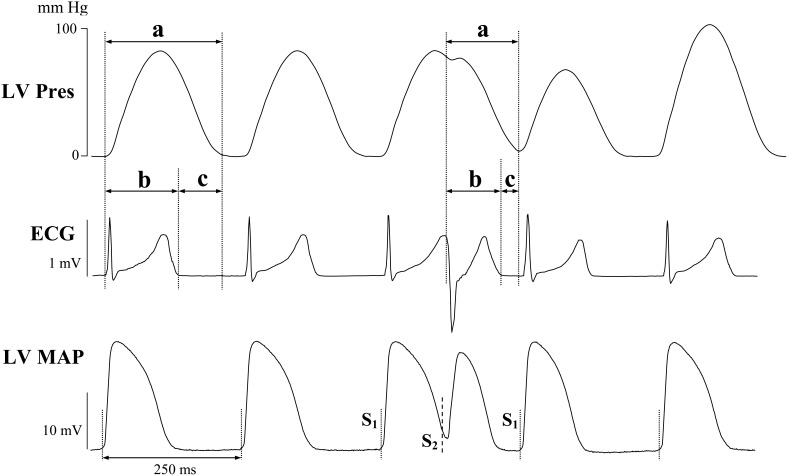
Assessments of durations of mechanical systole (a), QT interval (b) and electromechanical window (c) during steady-state pacing and extrasystolic stimulation in perfused guinea-pig heart. Basal representative recordings of left ventricular (LV) developed pressure (Pres), volume-conducted ECG, and LV epicardial monophasic action potentials (MAP) are shown. The dashed lines on the MAP trace in this figure, and Figures 3 and 4 indicate the moments of regular stimulus (S_1_) and premature stimulus (S_2_) application.

The duration of electromechanical systole was assessed by measuring the interval between the Q wave on ECG and the end of ventricular contraction on the LV pressure signal (Q-P_end_ interval; Figure 1). The EM window was calculated as the difference between the Q-P_end_ interval and the QT interval on ECG, as described previously [6], [8]–[9], [26]. The measurements were performed both during steady-state pacing and extrasystolic stimulation (Figure 1).

### Data collection and analysis

In total, 17 isolated, perfused heart preparations were used in this study, in order to assess electrophysiological effects produced by hypokalemia in guinea-pig (n = 10) and rabbit (n = 7) ventricles. The experiments were started with 30 min control normokalemic perfusion, followed by measurements of basal variables, and then switching to 30 min hypokalemic perfusion. During programmed stimulation, the electrophysiological parameters were determined in the last regular beat prior to extrastimulus application, and compared to those measured in the premature (S_2_-evoked) beat. The S_2_-evoked values were measured following extrasystolic stimulation applied at the shortest coupling stimulation interval that was either preceding refractoriness or preceding VT development (in hypokalemic heart preparations).

Data are expressed as means ± standard errors of the mean. Paired t-tests were used to compare samples forming two data sets. Fisher’s exact test was used to assess VT incidence in hypokalemic hearts. *P* values less than 0.05 were considered to be significant.

## Results

### Cardiac contractile function and arrhythmic susceptibility

In spontaneously beating heart preparations, hypokalemia had no effect on cardiac beating rate (Guinea-pig: Basal = 182±10 beats/min, Hypokalemia = 184±11 beats/min, *P* = 0.49; Rabbit: Basal = 143±11 beats/min, Hypokalemia = 144±11 beats/min, *P* = 0.89), LV developed pressure (Guinea-pig: Basal = 87±6 mm Hg, Hypokalemia = 85±7 mm Hg, *P* = 0.40; Rabbit: Basal = 79±8 beats/min, Hypokalemia = 82±4 mm Hg, *P* = 0.68), and coronary flow rate (Guinea-pig: Basal = 12±1 ml/min, Hypokalemia = 13±1 ml/min, *P* = 0.83; Rabbit: Basal = 40±4 ml/min, Hypokalemia = 42±3 ml/min, *P* = 0.61).

**Figure 2:**
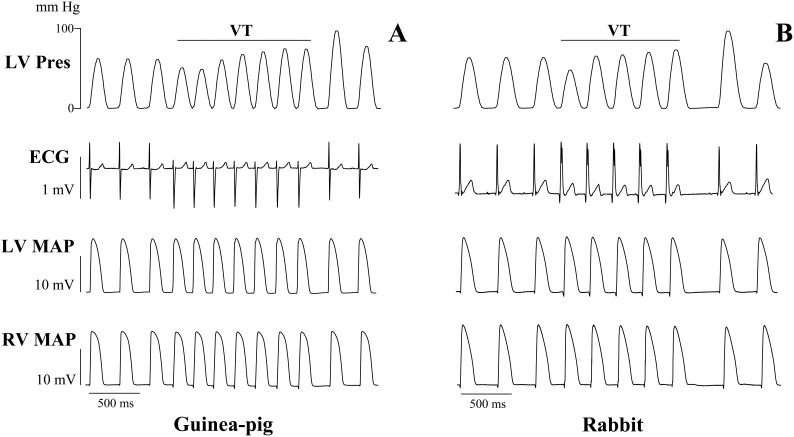
Representative episodes of monomorphic ventricular tachycardia (VT) recorded during hypokalemic perfusion in spontaneously beating guinea-pig and rabbit heart preparations. In each panel, simultaneous recordings of left ventricular (LV) developed pressure (Pres), volume-conducted ECG, and LV and right ventricular (RV) epicardial monophasic action potentials (MAP) are shown.

**Figure 3:**
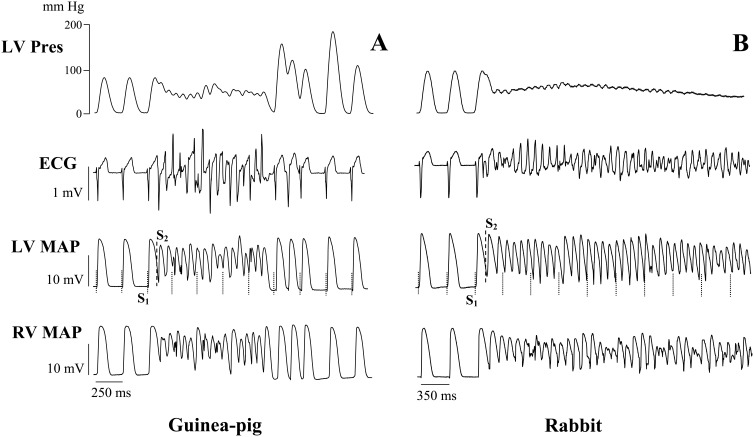
Representative episodes of torsade de pointes induced by programmed ventricular stimulation in hypokalemic guinea-pig and rabbit heart preparations. Panels A and B are arranged as described in the legend for Figure 2.

No episodes of spontaneous or programmed stimulation-evoked VT were observed during control normokalemic perfusion. Nevertheless, arrhythmic susceptibility was markedly increased during hypokalemia. Multiple ventricular ectopic beats were recorded during 30 min hypokalemic perfusion, both in guinea-pig and rabbit hearts. The short runs of monomorphic VT were observed in 7 out of 10 (70%) guinea-pig heart preparations (*P* = 0.003), and in 5 out of 7 (71%) rabbit heart preparations (*P* = 0.02) exposed to hypokalemia (Figure 2). During programmed LV stimulation (Figure 3), an extrastimulus application in the late repolarization phase immediately upon recurrence of ventricular excitability (i.e., at S_1_–S_2_ intervals exceeding ERP by 5–10 ms) was found to induce torsade de pointes in 8 out of 10 (80%) hypokalemic guinea-pig hearts (*P* = 0.0007), and in 5 out of 7 (71%) rabbit hearts (*P* = 0.02). In guinea-pig hearts, the episodes of torsade de pointes were short-lasting (0.5–2.0 s) and self-terminating. In rabbits, the tachyarrhythmia precipitated into sustained ventricular fibrillation in 3 out of 5 (60%) heart preparations with inducible torsade de pointes.

### QT interval, ventricular systole, and electromechanical window during S_1_–S_1_ pacing and extrasystolic stimulation

Table 1 shows QT intervals, Q-P_end_ intervals, and EM window values determined at baseline and upon hypokalemic perfusion in regular and extrasystolic beats evoked by programmed stimulation. During steady-state pacing, hypokalemia prolonged the QT interval in guinea-pig and rabbit hearts, but had no effect on Q-P_end_ interval. Consequently, the EM window was reduced in hypokalemic heart preparations (Table 1).

**Table 1:** Basal and hypokalemic QT intervals, Q-P_end_ intervals, and electromechanical window values determined during steady-state pacing and extrasystolic stimulations in guinea-pig and rabbit hearts.

	S_1_-beat		S_2_-beat	
	Basal	Hypokalemia	Basal	Hypokalemia
**Guinea-pig**				
QT interval	133±2	139±2*	105±4#	116±2*
Q-P_end_ interval	218±2	216±3	149±3#	153±3
Electromechanical window	85±3	77±3*	44±3#	37±3*
**Rabbit**				
QT interval	175±5	192±6*	165±5#	189±6*
Q-P_end_ interval	293±11	285±8	240±10#	244±7
Electromechanical window	118±10	93±7*	75±7#	55±3*

All parameters are given in ms. S_1_-evoked values were measured during LV epicardial pacing at a cycle length of 250 ms in guinea-pig hearts, and at 350 ms in rabbit hearts. Basal S_2_-evoked values were measured upon extrasystolic excitation with a coupling interval exceeding ERP by 5 ms. In hypokalemia, S_2_-evoked values were measured at the shortest effective coupling interval producing no tachyarrhythmia (Guinea-pig: S_1_–S_2_ = 105±2 ms; Rabbit: S_1_–S_2_ = 124±8 ms).

*P<0.05, hypokalemia vs. basal.

# *P*<0.05, basal S_2_-beat vs. basal S_1_-beat.

In basal conditions, extrasystolic stimulations applied over the late repolarization phase were associated with decreased QT interval, Q-P_end_ interval, and EM window, when compared to values determined in preceding S_1_ beats (Figure 1 and Table 1). Hypokalemia prolonged the QT interval in S_2_-evoked beats, while producing no effect on Q-P_end_ interval (Table 1). These changes translated to a reduced duration of EM window in S_2_-evoked beats (Table 1).

Importantly, in hypokalemic hearts, the prolonged QT interval nevertheless remained much shorter than the Q-P_end_ interval both in regular and extrasystolic beats (Table 1), thereby contributing to the positive EM window values (Regular beats: Guinea-pig = 77±3 ms, Rabbit = 93±7 ms; Extrasystolic beats: Guinea-pig = 37±3 ms, Rabbit = 55±3 ms).

### Ventricular conduction, effective refractory periods, and excitation wavelength index

In S_1_–S_1_ paced heart preparations, hypokalemia had no effect on mean LV activation time (Guinea-pig: Basal = 6.5±0.5 ms, Hypokalemia = 7.1±0.5 ms, *P* = 0.1; Rabbit: Basal = 12.6±1.3 ms, Hypokalemia = 13.7±1.4 ms, *P* = 0.68), but increased the mean RV activation time (Guinea-pig: Basal = 12.4±0.7 ms, Hypokalemia = 14.7±0.7 ms, *P* = 0.02; Rabbit: Basal = 26.3±2.2 ms, Hypokalemia = 30.3±1.5 ms, *P* = 0.02). Consequently, the LV-to-RV conduction time was increased upon hypokalemic perfusion from 5.9±0.5 ms to 7.6±0.6 ms (*P* = 0.03) in guinea-pig hearts, and from 13.7±1.5 ms to 16.6±1.6 ms (*P* = 0.04) in rabbit hearts.

Effective refractory periods were reduced in hypokalemic heart preparations from guinea-pig (Basal = 96±3 ms, Hypokalemia = 90±3 ms, *P* = 0.01) and rabbits (Basal = 118±5 ms, Hypokalemia = 105±4 ms, *P* = 0.04).

Reduced effective refractory periods in association with prolonged LV-to-RV conduction times contributed to decreased values of the excitation wavelength index in guinea-pig hearts (Basal = 16±2, Hypokalemia = 12±2, *P* = 0.01) and rabbit hearts (Basal = 9±1, Hypokalemia = 6±1, *P* = 0.02).

### Spatial variability in repolarization time

**Figure 4:**
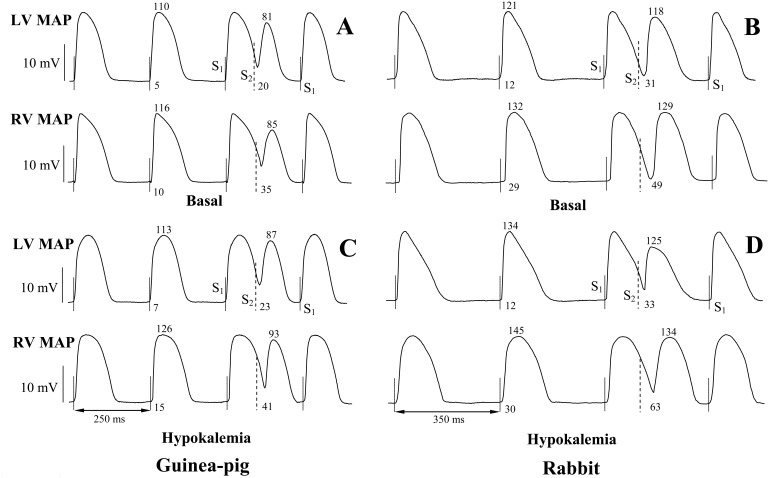
Representative left ventricular (LV) and right ventricular (RV) epicardial monophasic action potential (MAP) recordings obtained during programmed ventricular stimulation in basal conditions and following hypokalemic perfusion in guinea-pig and rabbit heart preparations. In each set of recordings, the numbers above the MAP trace indicate action potential duration (APD_90_) (ms), and the numbers under the MAP trace indicate the activation time values (ms) measured in regular beats (the second MAP in each trace) and extrasystolic beats (the fourth MAP in each trace). In basal recordings (panels A and B), note a greater action potential duration at RV compared to LV epicardium, and an increase in activation time along with APD_90_ shortening in S_2_-evoked beats as compared to preceding regular beats. Also note that hypokalemia (panels C and D) prolongs action potential duration, and increases the LV-to-RV difference in activation time, indicating conduction slowing.

**Figure 5:**
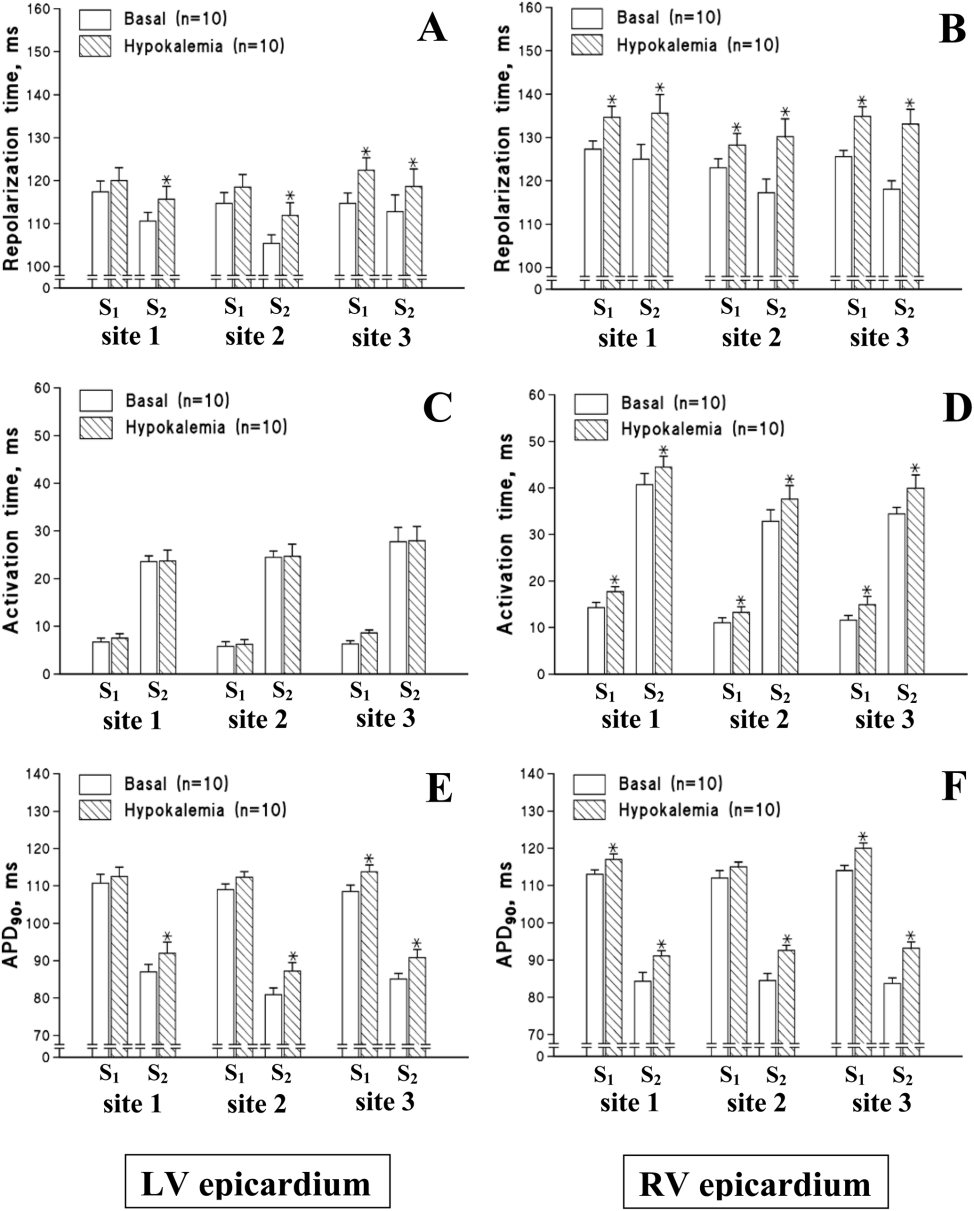
Effects of hypokalemia on the local repolarization time and its components, the activation time and action potential duration (APD_90_), determined at distinct left ventricular (LV) and right ventricular (RV) epicardial recording sites during steady-state pacing (S_1_) and extrasystolic stimulation (S_2_) in guinea-pig heart preparations. The measurements were taken from three LV recording sites (site 1 is the lateral LV wall, site 2 is the anterior LV wall, and site 3 is the posterior LV wall) and three RV recording sites (site 1 is the posterior RV wall, site 2 is the anterior RV wall, and site 3 is the lateral RV wall), while implementing the programmed stimulation protocol shown in Figure 4. *P<0.05 vs. corresponding basal value.

**Figure 6:**
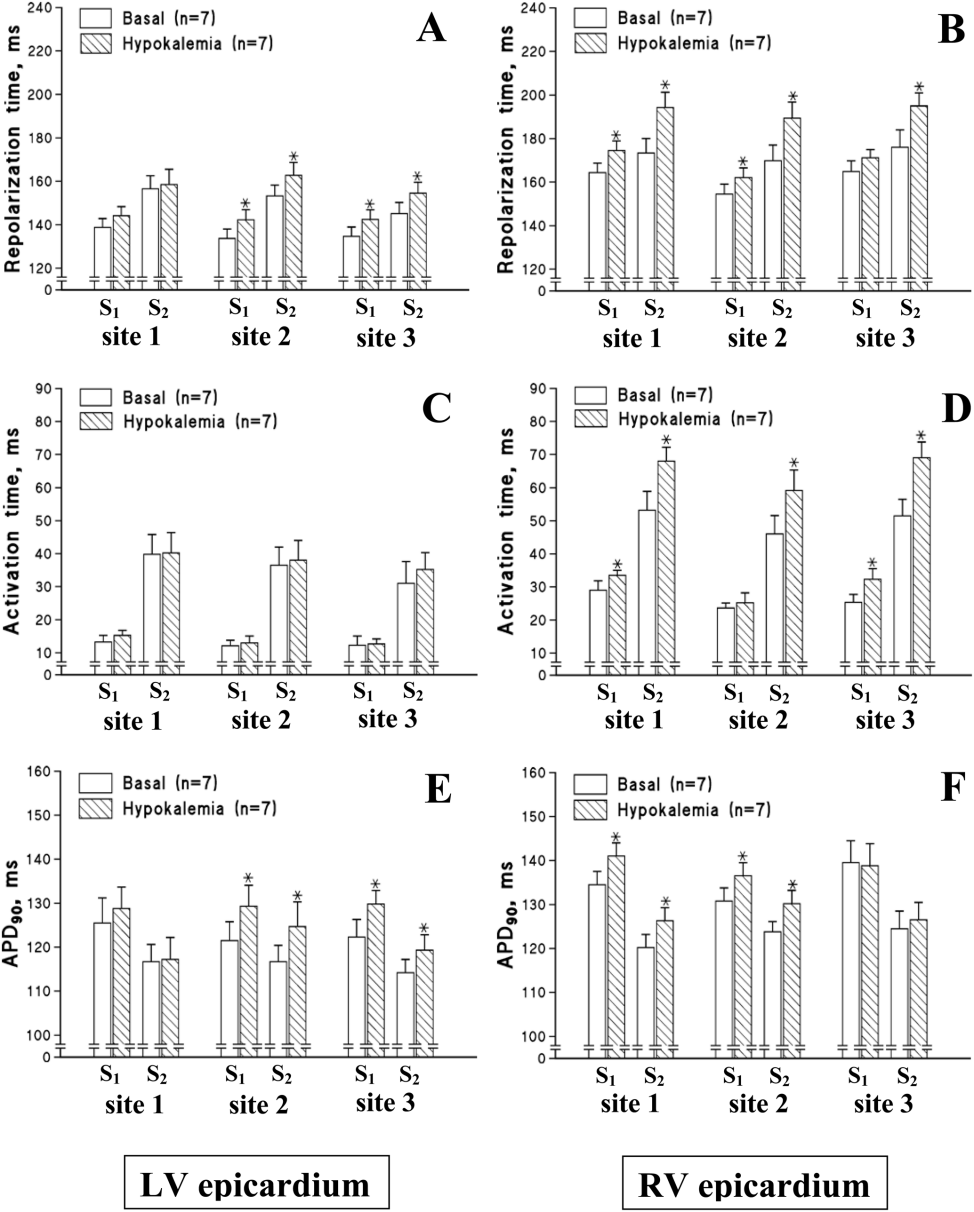
Effects of hypokalemia on the local repolarization time and its components, the activation time and action potential duration (APD_90_), determined at distinct left ventricular (LV) and right ventricular (RV) epicardial recording sites during steady-state pacing (S_1_) and extrasystolic stimulation (S_2_) in rabbit heart preparations. The location of individual epicardial recording sites in LV and RV chamber is indicated in the legend for Figure 5. *P<0.05 vs. corresponding basal value.

Figure 4 shows the representative LV and RV monophasic action potentials recorded upon programmed ventricular stimulation before and after hypokalemic perfusion in guinea-pig and rabbit hearts, and Figures 5 and 6 show composite data illustrating changes in repolarization times and its constituents (the activation time and APD_90_) determined at individual epicardial recording sites.

In basal conditions, the mean repolarization time value measured from six epicardial recording sites during S_1_–S_1_ pacing was found to be 120±2 ms in guinea-pig hearts, and 148±5 ms in rabbit hearts. The standard deviation of the mean repolarization time value was 6.9±0.4 ms in guinea-pig hearts, and 13.9±1.0 ms in rabbit hearts. In both animal species, transepicardial variability in repolarization time was attributed to longer action potential duration in the RV compared to the LV chamber (Guinea-pig: Mean RV APD_90_ = 114±2 ms, Mean LV APD_90_ = 109±2 ms, *P* =  = 0.04; Rabbit: Mean RV APD_90_ = 135±5 ms, Mean LV APD_90_ = 123±5 ms, *P* = 0.04), and to later RV than LV activation upon LV epicardial pacing (Guinea-pig: Mean LV activation time = 6.5±0.5 ms, Mean RV activation time = 12.4±0.7 ms, *P*<0.001; Rabbit: Mean LV activation time = 12.6±1.3 ms, Mean RV activation time = 26.3±2.2 ms, *P*<0.001). Extrasystolic stimulations were associated with conduction slowing, as evidenced by increased activation time in S_2_- as compared to S_1_-evoked beats, and shortened APD_90_ (the restitution effect) in guinea-pig hearts (Figure 4A and Figure 5, panels C–F) and rabbit hearts (Figure 4B and Figure 6, panels C–F).

In hypokalemic hearts, the mean repolarization time value from six epicardial recording sites was increased during both regular pacing (Guinea-pig: Basal = 120±2 ms, Hypokalemia = 127±2 ms, *P* = 0.03; Rabbit: Basal = 148±5 ms, Hypokalemia = 156±4 ms, *P* = 0.04) and extrasystolic stimulation (Guinea-pig: Basal = 115±2 ms, Hypokalemia = 124±3 ms, *P* = 0.01; Rabbit: Basal = 162±6 ms, Hypokalemia = 176±7 ms, *P* = 0.04). Throughout the ventricular epicardium, the prolongation of repolarization time was highly non-uniform. For example, in hypokalemic guinea-pig hearts, the minimal effect on repolarization time (S_1_-beat: +3 ms; S_2_-beat: +5 ms) was produced in the LV lateral wall (site 1 in Figure 5A), whereas the maximal effect (S_1_-beat: +9 ms; S_2_-beat: +15 ms) was evoked in the RV lateral wall (site 3 in Figure 5B). Consequently, the spatial variability of repolarization time was increased in hypokalemic hearts during regular pacing (Standard deviation of the mean repolarization time: Basal = 6.9±0.4 ms, Hypokalemia = 8.9±0.6 ms, *P* = 0.01) and extrasystolic stimulation (Standard deviation of the mean repolarization time: Basal = 9.9±1.0 ms, Hypokalemia = 12.8±1.0 ms, *P* = 0.04).

Non-uniform changes in repolarization time have also been observed in hypokalemic rabbit hearts, wherein the minimal effect (S_1_-beat: +5 ms; S_2_-beat: +2 ms) was elicited in the LV lateral wall (site 1 in Figure 6A), whereas the maximal effect (S_1_-beat: +10 ms; S_2_-beat: +21 ms) was determined in the RV posterior wall (site 1 in Figure 6B). These changes translated to amplified transepicardial variability of repolarization time both during regular pacing (Standard deviation of the mean repolarization time: Basal = 13.9±1.0 ms, Hypokalemia = 18.1±1.2 ms, *P* = 0.02) and extrasystolic stimulation (Standard deviation of the mean repolarization time: Basal = 16.9±1.4 ms, Hypokalemia = 22.5±2.7 ms, *P* = 0.04).

### Epicardial activation times and action potential durations

Increased spatial non-uniformities in repolarization time in hypokalemic hearts were attributed to dissimilar lengthening of activation time and action potential duration at individual epicardial recording sites. During both regular pacing and extrasystolic stimulations, the activation time was increased by hypokalemia in RV recording sites, but remained unchanged at LV recording sites, in both guinea-pig hearts (Figure 5, C–D) and rabbit hearts (Figure 6, C–D). Regarding the effects on action potential duration, an increase in APD_90_ in S_1_–S_1_ paced guinea-pig heart preparations was observed in only 3 out of 6 epicardial sites, including the LV posterior wall (site 3 in Figure 5E), the RV posterior wall (site 1 in Figure 5F), and the RV lateral wall (site 3 in Figure 5 F). During extrasystolic stimulations, hypokalemia evoked APD_90_ lengthening in all 6 recording sites, but the effect was variable, and ranged from +5 ms (LV lateral wall, site 1 in Figure 5E) to +10 ms (RV lateral wall, site 3 in Figure 5F).

Likewise, spatially non-uniform changes in epicardial action potential duration were observed in hypokalemic rabbit hearts (Figure 6, E–F). During both steady-state pacing and extrasystolic stimulations, hypokalemia produced no effect in the LV lateral wall (site 1 in Figure 6E) and RV lateral wall (site 3 in Figure 6F), whilst prolonging APD_90_ in the LV anterior and posterior walls (site 2 and site 3, respectively, in Figure 6E), and in the RV anterior and posterior walls (site 2 and site 1, respectively, in Figure 6F).

## Discussion

### Main findings

This study suggests that hypokalemia-induced arrhythmogenicity may not be accounted for by the reversed relationships between the duration of electrical and mechanical systole, which have been reported to occur in other experimental models of electrical instability [6], [8]–[9]. Indeed, although hypokalemia was found to prolong repolarization and increase the occurrence of tachyarrhythmia in perfused guinea-pig and rabbit hearts, the duration of mechanical systole remained invariably longer compared to the QT interval, thereby contributing to the positive EM window, as assessed during both steady-state pacing and extrasystolic stimulations. Nevertheless, proarrhythmic effects of hypokalemia were associated with slowed LV-to-RV conduction and shortened effective refractory periods, which translated to a reduced excitation wavelength index. Furthermore, hypokalemia evoked non-uniform prolongation of repolarization time at distinct epicardial recording sites, which resulted in amplified spatial repolarization gradients. These findings therefore suggest that in hypokalemic hearts, the abnormal changes in ventricular conduction times, refractoriness, excitation wavelength, and repolarization gradients are more important mechanistic determinants of arrhythmic substrate, as compared to the changes in EM window.

### Electromechanical window, arrhythmic susceptibility, and hypokalemia

The negative EM window (wherein QT interval exceeds the duration of mechanical systole) appears to be a clinically relevant phenomenon, which has been observed in different cardiovascular conditions including mitral leaflet prolapse [4], coronary artery disease [3], and inherited long QT syndrome [5]. Likewise, a positive difference between the duration of mechanical systole and QT interval seen in normal human subjects, may be reversed upon acute adrenergic stimulation produced by β-adrenoreceptor agonist infusion [27] or intensive physical exercise [2], [5]. Importantly, in patients with healed myocardial infarction, the long-term survival rate was found to be 2.6-fold lower in a patient subgroup with a negative EM window, thereby indicating that this parameter may be used to predict the mortality risk in coronary artery disease [3]. More recently, these clinical findings were substantiated by animal studies, which demonstrate that the negative EM window markedly increases susceptibility to life-threatening ventricular tachyarrhythmia, such as torsade de pointes [6], [8]–[9]. In a canine model of long QT syndrome, a negative EM window was found to be a prerequisite for VT initiation [6]. In anesthetized guinea-pigs, administration of drugs with known high proarrhythmic potential (i.e., quinidine, haloperidol, and domperidone) was found to induce a negative EM window, whereas safe antiarrhythmics such as amiodarone, verapamil and diltiazem produced no effect [8]–[9]. Increased electrical instability in the presence of the negative EM window is thought to be attributed to abnormal Ca^2+^ handling, wherein Ca^2+^ can continue to enter into the cardiac cells and trigger sarcoplasmic reticulum Ca^2+^ release, after completing mechanical contraction. This leads to Ca^2+^ overload, thus facilitating both early and delayed after-depolarizations, which are known to play a role in initiating VT [7], [9].

The role of the negative EM window in the mechanism of drug-induced proarrhythmia was nevertheless challenged in study by Laursen et al. (2011) [26], who showed that in perfused mini-pig and dog hearts, the EM window remained positive even in the setting wherein a blockade of the delayed rectifier K^+^ current (which prolongs QT interval) was combined with β-adrenoreceptor agonist challenge (which abbreviates mechanical systole). However, no attempts have been previously made to examine whether the mismatch between the duration of electrical and mechanical systole may contribute to arrhythmic substrate in the setting of hypokalemia, the most common electrolyte abnormality seen in cardiac patients [14]–[17], [28].

The ALLHAT trial showed that hypokalemia may be found in 13% of hypertensive patients treated with diuretics [28]. Hypokalemia promotes QT interval lengthening and electrical instability, which may be exaggerated in patients with hereditary long QT syndrome [14]. In recent clinical studies, low serum K^+^ levels were associated with increased mortality in cardiac patients [15]–[17]. Arrhythmogenic effects produced by hypokalemia were also widely replicated in animal models [29]–[31].

Importantly, hypokalemia-induced arrhythmogenicity may potentially be linked to the reverse relationships between the QT interval and mechanical systole. First, hypokalemia has been shown to reduce the magnitude of repolarizing K^+^ currents such as *I*_Kr_, the rapid component of the delayed rectifier, and *I*_K1_, the inward rectifier [29], [32], which translates to prolongation of the QT interval on ECG. Furthermore, hypokalemia may affect intracellular Ca^2+^ handling via mechanisms related to inhibition of Na^+^-K^+^ pump and subsequent stimulation of the reversed mode of the Na^+^-Ca^2+^ exchange [33]–[34]. These changes would likely result in increased ventricular contraction, and abbreviated mechanical systole. Taken together, the aforementioned effects of hypokalemia may be expected to contribute to the negative EM window. This notion, however, is not supported by the outcomes of the present study. Although hypokalemia was found to moderately prolong the QT interval and reduce the time difference between the end of electrical systole and the end of ventricular contraction, the EM window always remained in the range of positive values both during steady-state pacing and extrasystolic stimulations (Table 1).

### Epicardial conduction, refractoriness, and excitation wavelength

The excitation wavelength refers to the distance travelled by the depolarization wavefront during the refractory period [35]. As ventricular conduction time is the inverse correlate of conduction velocity, the excitation wavelength may be indirectly assessed by calculating the ratio between the effective refractory period and conduction time [23]–[25]. In this study, hypokalemia was found to produce conduction slowing, as evidenced by increased mean LV-to-RV conduction delay, an effect that is presumably accounted for by membrane hyperpolarization and the enhanced ventricular excitation threshold typically seen in hypokalemic ventricular muscle [36]. The LV effective refractory period was reduced by hypokalemia both in guinea-pig and rabbit hearts, which may be attributed, at least in part, to hypokalemia effects on the recovery of Na^+^ channels from inactivation. In support of this argument, the recurrence of excitability during the repolarization phase was found to occur at less negative membrane potentials in hypokalemic guinea-pig papillary muscle, and this effect was abolished by Na^+^ channel blocker [37].

Reduced effective refractory period, in association with prolonged interventricular conduction time, contributed to a decreased value of the excitation wavelength index in hypokalemic guinea-pig and rabbit hearts in the present study. This change may set a stage for tachyarrhythmia, because a reduced excitation wavelength allows re-entry within a smaller mass of cardiac tissue to be sustained [35]. Consistently, the runs of torsade de pointes were inducible by programmed LV stimulation in 70–80% of hypokalemic heart preparations used in this study (Figure 3).

### Spatial variability in repolarization time

The expression levels and current density of outward K^+^ currents are variable throughout the ventricular epicardium [38]–[40], which contributes to spatial non-uniformities in action potential duration. In this study, the basal APD_90_ values measured on steady-state pacing were found to be greater in RV compared to the LV chamber both in guinea-pig and rabbit hearts. Importantly, spatial heterogeneities in the distribution of K^+^ currents may also contribute to the dissimilar prolongation of repolarization at distinct myocardial regions, as observed in the present study (Figures 5 and 6). These changes translated to amplified spatial repolarization gradients, as indicated by the increased standard deviation of the mean repolarization time value determined at six epicardial recording sites. Enhanced variability in the distribution of repolarization time across the ventricular epicardium may set a stage for unidirectional conduction block and re-entry, thus contributing to proarrhythmic effects produced by hypokalemia.

### Limitations

In this study, although hypokalemia-induced arrhythmogenicity was not associated with a negative EM window, the positive value of EM window determined in guinea-pig and rabbit hearts at baseline was nevertheless reduced upon hypokalemic perfusion, an effect that can be ascribed to prolongation of the QT interval (Table 1). Therefore, hypokalemia would likely facilitate the induction of a negative EM window when it is applied concomitantly with other factors that produce a mismatch between the electrical and mechanical activities, either owing to prolongation of the QT interval (e.g., class III antiarrhythmic agents) or shortening of the duration of LV contraction (e.g., β-adrenoreceptor agonist challenge). However, in the present study, hypokalemia itself was found to be a sufficiently powerful arrhythmic challenge that promoted VT in majority of heart preparations used, and hence electrophysiological effects produced by a combination of hypokalemia and other proarrhythmic factors were not tested.

The size of the guinea-pig and rabbit hearts is much smaller than the human heart, meaning that the ability to sustain re-entrant cardiac arrhythmia is less in these animal species. This difference may imply that the same degree of hypokalemia (2.5 mM K^+^) would be associated with greater occurrence and duration of cardiac arrhythmias in human patients, as compared to those reported in the present study.

### Conclusions

In summary, this study suggests that increased propensity to VT in hypokalemic guinea-pig and rabbit heart preparations is not accounted for by the negative EM window, and is rather attributed to slowed interventricular conduction, shortened LV effective refractory period, reduced excitation wavelength index, and amplified variability in distribution of repolarization time throughout the ventricular epicardium.

## Supporting Information

Figure S1 **Location of the monophasic action potential recording electrodes in perfused heart preparations.** Monophasic action potential recording electrodes were attached to the left ventricular (LV) epicardium (solid circles in panels A and C) and the right ventricular (RV) epicardium (open circles in panels B and C). Panel C shows that in each ventricular chamber, the electrodes were placed in anterior ventricular wall (AW), lateral ventricular wall (LW), and posterior ventricular wall (PW). LAD is the left anterior descending coronary artery.
(PDF)
