# Supplementary figures and images for: Impact of Hypokalemia on Electromechanical Window, Excitation Wavelength and Repolarization Gradients in Guinea-Pig and Rabbit Hearts
Source: PLoS One. 2014 Aug 20;9(8):e105599. doi: 10.1371/journal.pone.0105599 (PMC4139393; doi:10.1371/journal.pone.0105599)

**LV front view**

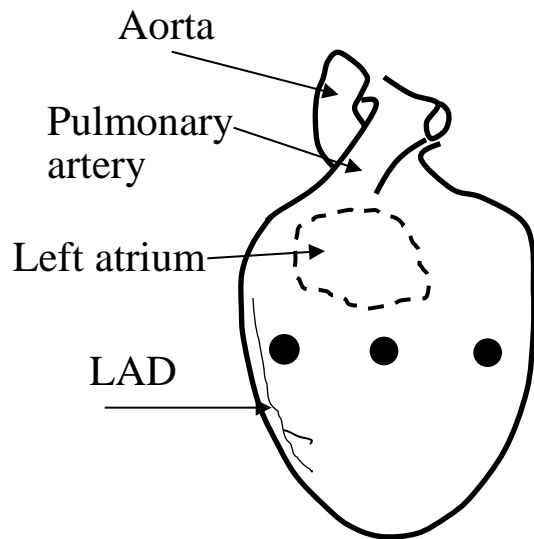

**A**

**RV front view**

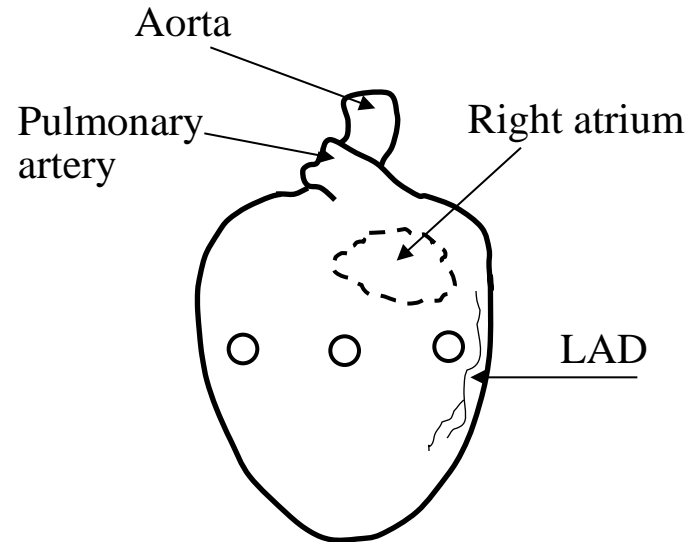

**B**

**Top cross-sectional view**

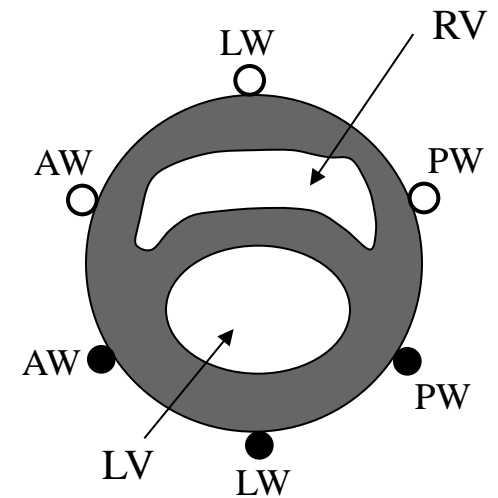

**C**

**Supplemental Figure 1**

Supplement: Figure S1 — Location of the monophasic action potential recording electrodes in perfused heart preparations. Monophasic action potential recording electrodes were attached to the left ventricular (LV) epicardium (solid circles in panels A and C) and the right ventricular (RV) epicardium (open circles in panels B and C). Panel C shows that in each ventricular chamber, the electrodes were placed in anterior ventricular wall (AW), lateral ventricular wall (LW), and posterior ventricular wall (PW). LAD is the left anterior descending coronary artery. (PDF) [file pone.0105599.s001.pdf]
